# Purification and Characterization of Antioxidant Peptides Derived from Protein Hydrolysate of the Marine Bivalve Mollusk *Tergillarca granosa*

**DOI:** 10.3390/md17050251

**Published:** 2019-04-27

**Authors:** Xiu-Rong Yang, Yi-Ting Qiu, Yu-Qin Zhao, Chang-Feng Chi, Bin Wang

**Affiliations:** 1 National and Provincial Joint Laboratory of Exploration and Utilization of Marine Aquatic Genetic Resources, National Engineering Research Center of Marine Facilities Aquaculture, School of Marine Science and Technology, Zhejiang Ocean University, Zhoushan 316022, China; yxr1948008999@163.com; 2 Zhejiang Provincial Engineering Technology Research Center of Marine Biomedical Products, School of Food and Pharmacy, Zhejiang Ocean University, Zhoushan 316022, China; qytdezh@icloud.com (Y.-T.Q.); zhaoy@hotmail.com (Y.-Q.Z.)

**Keywords:** blood cockle (*Tegillarca granosa*), protein hydrolysate, bioactive peptide, antioxidant activity

## Abstract

In this report, protein hydrolysate (TGH) of blood cockle (*Tegillarca granosa*) was prepared using a two-enzyme system (Alcalase treatment for 1.5 h following Neutrase treatment for 1.5 h). Subsequently, six antioxidant peptides were isolated from TGH using ultrafiltration and chromatography methods, and their amino acid sequences were identified as EPLSD, WLDPDG, MDLFTE, WPPD, EPVV, and CYIE with molecular weights of 559.55, 701.69, 754.81, 513.50, 442.48, and 526.57 Da, respectively. In which, MDLFTE and WPPD exhibited strong scavenging activities on DPPH radical (EC_50_ values of 0.53 ± 0.02 and 0.36 ± 0.02 mg/mL, respectively), hydroxy radical (EC_50_ values of 0.47 ± 0.03 and 0.38 ± 0.04 mg/mL, respectively), superoxide anion radical (EC_50_ values of 0.75 ± 0.04 and 0.46 ± 0.05 mg/mL, respectively), and ABTS cation radical (EC_50_ values of 0.96 ± 0.08 and 0.54 ± 0.03 mg/mL, respectively). Moreover, MDLFTE and WPPD showed high inhibiting ability on lipid peroxidation. However, MDLFTE and WPPD were unstable and could not retain strong antioxidant activity at high temperatures (>80 °C for 0.5 h), basic pH conditions (pH > 9 for 2.5 h), or during simulated GI digestion. In addition, the effect of simulated gastrointestinal digestion on TGP4 was significantly weaker than that on MDLFTE. Therefore, MDLFTE and WPPD may be more suitable for serving as nutraceutical candidates in isolated forms than as food ingredient candidates in functional foods and products.

## 1. Introduction

Toxic reactive oxygen species (ROS) induced by oxidative stress destroy structures of some functional biomacromolecules including DNA, proteins, and membrane lipids, which further lead to some chronic diseases, such as liver damage, type 2 diabetes, asthma, neurodegenerative diseases, and arthritis [1,2,3]. In addition, oxidative deterioration produces some off-flavors and harmful lipid metabolites, which negatively influence the food quality and somatic functions [2,4]. Therefore, eliminating superfluous ROS is important for keeping cellular homeostasis. At present, people often use synthetic antioxidants to prevent and intervene ROS damage, but the negative effects of synthetic antioxidants, including liver damage and carcinogenesis, limit their scope of usage and dosage [5,6]. Therefore, looking for nontoxic natural antioxidants is of great significance for food, cosmetic, and pharmaceutical industries.

Bioactive peptides (BPs), which in general range from 2 to 20 amino acids in size, are fragments of proteins and become active when released during enzymatic hydrolysis, gastrointestinal transit, and fermentation [7,8]. Recently, BPs released from proteins of aquatic products and their by-products have been demonstrated to show multiple biological properties, including hypolipidemic, angiotensin-converting-enzyme (ACE) inhibitory, antioxidative, anti-diabetic, and immunomodulatory activities [9,10,11,12]. Furthermore, there is growing interest worldwide in discovering antioxidant peptides (APs) from food proteins and applying them in functional foods and nutraceuticals due to their biological and nutritional properties [5]. HPLDSLCL from protein hydrolysate of ark shell (*Scapharca subcrenata*) showed potent 2,2-diphenyl-1-picrylhydrazyl (DPPH) and 2,20-azino-bis-3-ethylbenzothiazoline-6-sulfonic acid (ABTS) cation radical scavenging activities, reducing power, and oxidative inhibition on copper-catalyzed human low-density lipoprotein [13]. AAVPSGASTGIYEALELR (molecular weight (MW) of 1805.03 Da) and NPLLEAFGNAK (MW of 1173.34 Da) isolated from gonad hydrolysate of purple sea urchin can activate DAF-16 pathway and increase the expression of DAF-16 target genes, which further reduce ROS level and the expression of heat shock protein-16.2 and superoxide dismutase-3 (SOD-3) in oxidation-damaged nematodes [14]. SDITRPGNM from protein hydrolysate of *Palmaria palmata* has potential applications as a food preservative and health enhancing ingredient because of its high reducing power and oxygen radical absorbance capacity [4]. Wong et al. reported that WAFAPA and MYPGLA from blue-spotted stingray showed stronger inhibiting ability than that of carnosine on H_2_O_2_-induced lipid oxidation and could protect plasmid DNA and proteins from oxidative damage induced by Fenton′s reagent. In addition, EC_50_ (half elimination ratio) value of WAFAPA (8.33 mg/mL) on ABTS cation radical was lower than that of MYPGLA (12.88 mg/mL) [15]. Yang et al. reported that GADIVA and GAEGFIF from gelatin hydrolysate of skipjack tuna bone might serve as potential candidates in health-promoting food industries due to their strong antioxidant activities, including lipid peroxidation inhibition ability, radical scavenging activity, and reducing power [16]. Therefore, BPs isolated from seafoods and their by-products exhibit significant antioxidant activity in protecting living organism from oxidative damage.

Blood cockle (*Tegillarca granosa*), which resides along the estuarine and coastal regions of the Indo-Pacific, pertains to the *Arcidae* family, and is one significantly economic bivalve species [17]. In south provinces of China, blood cockle is an important and nutritious festival food and its large-scale aquaculture production has been practiced. In addition, blood cockles have been served as traditional medicine for regulating the secretion of human gastric acid and curing inflammation, stomachache, anemia, and cancers [17,18]. Furthermore, Han et al. reported that polypeptides (MW ranged between 1000 and 5000 Da) from *T. granosa* showed significant antitumor activity and could improve immune responses without significant toxic side effects, which suggested that those polypeptides might be suitable for combination therapy of cancer patients [19]. In our previous work, two APs including Trp-Pro-Pro (WPP) and Gln-Pro (QP) were isolated and identified from Neutrase hydrolysate of *T. granosa* protein, and WPP has the potential to serve as a natural antioxidant and anticancer agent for the nutraceutical and pharmaceutical industries because of its high radical scavenging activity and strong cytotoxicity toward PC-3, DU-145, H-1299, and HeLa cell lines [17]. However, past studies have not provided enough support for promoting the application of blood cockle in functional food and products. In order to make full use of these resources and look for nontoxic natural antioxidants, the aims of this work were to (i) prepare and characterize APs from protein hydrolysate of blood cockle (TGH) and (ii) evaluate the stability and in vitro antioxidant activity of the isolated APs.

## 2. Results and Discussion

### 2.1. Purification of APs from TGH

#### 2.1.1. Preparation and Fractionation of TGH

Defatted muscle of blood cockle was hydrolyzed under a two-enzyme system (Alcalase treatment for 1.5 h following Neutrase treatment for 1.5 h), and the degree of hydrolysis (DH) and yield of the resulted hydrolysate (referred to as TGH) were 19.32 ± 1.37% and 9.62 ± 0.86% (on the basis of defatted muscle), respectively. In addition, TGH could strongly scavenge DPPH radical with EC_50_ value of 3.55 ± 0.32 mg protein/mL, which was lower than those of protein hydrolysates from miiuy croaker muscle (<50% at 5 mg/mL) [2] and swim bladder (13.55 mg/mL) [11], bluefin leatherjacket head (15.98% at 10 mg/mL) [20], and tilapia skin (3.66 mg/mL) [21], but higher than those of protein hydrolysates of sea urchin gonad (0.945 mg/mL) [14] and salmon pectoral fin (1.63 mg/mL) [22].

TGH was further fractionized with MW Cut Off (MWCO) membranes of 3, 5, and 10 kDa, and four fractions including TGH-І (<3 kDa), TGH-II (3–5 kDa), TGH-III (5–10 kDa), and TGH-IV (>10 kDa) were prepared. The EC_50_ value of TGH-І on DPPH radical was 2.83 ± 0.17 mg protein/mL, which was significantly (*P* < 0.05) stronger than those of TGH (3.55 mg protein/mL), TGH-II (5.28 ± 0.35 mg protein/mL), TGH-III (7.58 ± 0.32 mg protein/mL), and TGH-IV (9.36 ± 0.54 mg protein/mL). TGH was composed of different chain length peptides. Short peptides are more accessible and trap the free radicals more easily [5,23]. The DPPH radical activity of TGH and its fractions were in accordance with previous literature that the antioxidant abilities of protein hydrolysates were negatively correlated with their average MW [24]. Therefore, TGH-І accounting for 13.24 ± 1.23% of TGH was selected for the subsequent chromatographic separation.

#### 2.1.2. Chromatography Isolation of APs from TGH-I

**Figure 1:**
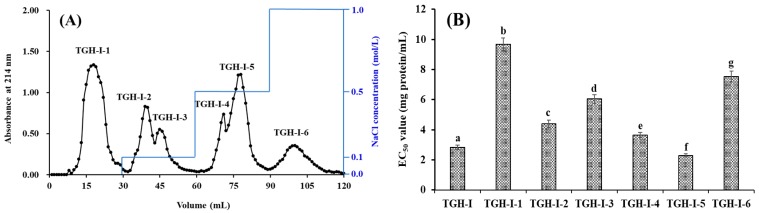
Elution profile of TGH-І in DEAE-52 cellulose anion-exchange chromatography (**A**) and EC_50_ values of TGH-1 and its fractions on DPPH radical (**B**). TGH-І-1 collected from 10 mL to 27 mL; TGH-І-2 collected from 34 mL to 42 mL; TGH-І-3 collected from 43 mL to 51 mL; TGH-І-4 collected from 65 mL 72 mL; TGH-І-5 collected from 73 mL to 85 mL; and TGH-І-6 collected from 92 mL to 110 mL. All data are expressed as mean ± standard deviation (SD, n = 3). a–g values with the same letters indicate no significant difference of different sample (*p* > 0.05).

As shown in Figure 1A, six fractions (TGH-І-1 to TGH-І-6) were separated from TGH-І using a DEAE-52 cellulose column. In which, TGH-І-1 was eluted using deionized water (DW); TGH-І-2 and TGH-І-3 were eluted using 0.1 M NaCl; TGH-І-4 and TGH-І-5 were eluted using 0.5 M NaCl; and TGH-І-6 were eluted using 1.0 M NaCl. EC_50_ values of TGH-І and its six fractions on DPPH radical were showed in Figure 1B, and the data demonstrated that TGH-І-5 with EC_50_ value of 2.29 ± 0.11 mg protein/mL showed significantly stronger DPPH radical scavenging activity than those of TGH-I (2.82 ± 0.16 mg protein/mL), TGH-І-1 (9.68 ± 0.42 mg protein/mL), TGH-І-2 (4.39 ± 0.25 mg protein/mL), TGH-І-3 (6.04 ± 0.28 mg protein/mL), TGH-І-4 (3.65 ± 0.17 mg protein/mL), and TGH-I-6 (7.53 ± 0.36 mg protein/mL) (*p* < 0.05). Ion exchange resins with different kinds of ionic forms and particle sizes are applied for the preparation of charged compounds and wiping off ionic molecules [17]. Functional molecules with negatively charges can bind to positively charged resins on Van der Waals forces and be separated from mixture solutions [25]. Then, TGH-І-5 with a yield of 10.43 ± 0.76% of TGH-І will be used for the subsequent experiment.

**Figure 2:**
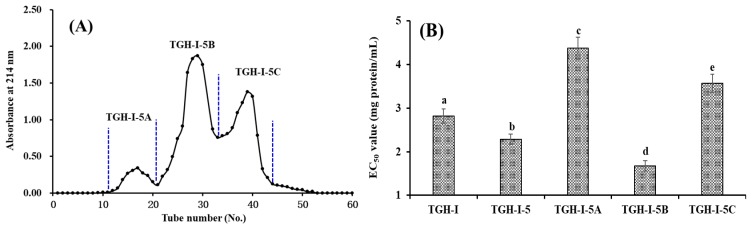
Elution profile of TGH-І-5 in Sephadex G-25 column (**A**) and EC_50_ values of TGH-1-5 and its fractions on DPPH radical (**B**). TGH-1-5A collected from No. 11 to 21; TGH-1-5B collected from No. 22 to 32; and TGH-1-5C collected from No. 33 to 43. All data are expressed as mean ± SD (n = 3). a–e values with the same letters indicate no significant difference of different sample (*p* > 0.05).

**Figure 3:**
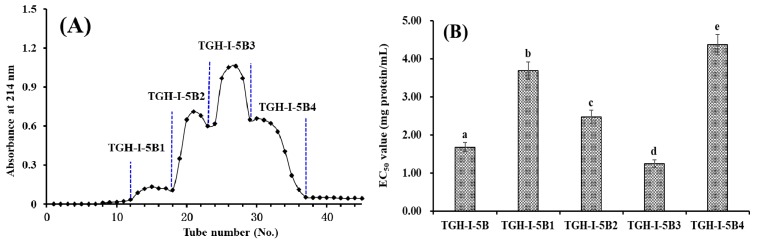
Elution profile of TGH-І-5B in Sephadex G-15 column (**A**) and EC_50_ values of TGH-1-5B and its fractions on DPPH radical (**B**). TGH-1-5B1 collected from No. 12 to 18; TGH-1-5B2 collected from No. 19 to 23; TGH-1-5B3 collected from No. 24 to 29; and TGH-1-5B4 collected from No. 30 to 37. All data are expressed as mean ± SD (n = 3). a–e values with same letters indicate no significant difference of different sample (*p* > 0.05).

TGH-І-5 was further fractionized into three fractions (TGH-І-5A, TGH-І-5B, and TGH-І-5C) using a Sephadex G-25 column (Figure 2A). The EC_50_ value of TGH-І-5B on DPPH radical was 1.68 ± 0.12 mg protein/mL, which was significantly (*p* < 0.05) lower than those of TGH-І-5 (2.29 ± 0.11 mg protein/mL), TGH-І-5A (4.38 ± 0.25 mg protein/mL), and TGH-І-5C (3.57 ± 0.21 mg protein/mL) (Figure 2B). Subsequently, TGH-І-5B accounting for 30.94 ± 2.62% of TGH-І-5 was separated into four fractions (TGH-І-5B1 to TGH-І-5B4) using Sephadex G-15 column (Figure 3A) and their antioxidant activity are presented in Figure 3B. The EC_50_ value of TGH-І-5B3 on DPPH radical was 1.25 ± 0.10 mg protein/mL, which was significantly (*p* < 0.05) higher than those of TGH-І-5B (1.68 ± 0.12 mg protein/mL), TGH-І-5B1 (3.69 ± 0.23 mg protein/mL), TGH-І-5B2 (2.47 ± 0.18 mg protein/mL), and TGH-І-5B4 (4.37 ± 0.26 mg protein/mL). Therefore, TGH-І-5B3 accounting for 18.62 ± 1.33% of TGH-І-5B was suitable for the following separation process.

**Figure 4:**
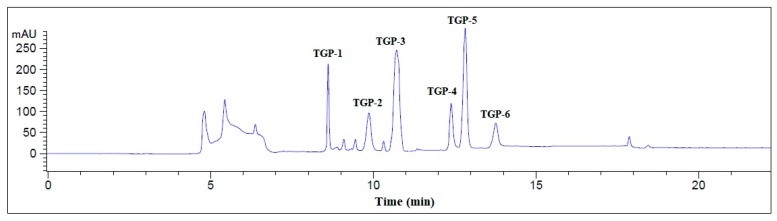
Elution profile of TGH-І-5B3 separated by RP-HPLC system on a Zorbax C-18 column (4.6 × 250 mm) from 0 to 25 min.

As shown in Figure 4, six APs with a retention time of 8.586 min (TGP-1), 9.831 min (TGP-2), 10.679 min (TGP-3), 12.394 min (TGP-4), 12.692 min (TGP-5), and 13.762 min (TGP-6) were isolated from TGH-І-5B3 using an HPLC system with a Zorbax C-18 column (Table 1), and the eluted peptides were collected separately on their chromatographic peaks and lyophilized for analysis of the amino acid sequences, antioxidant activities, and stability properties. In addition, the yields of six isolated APs were 40.52 ± 3.76 mg/100 g TGH (TGP-1), 9.43 ± 0.64 mg/100 g TGH (TGP-2), 23.46 ± 1.51 mg/100 g TGH (TGP-3), 12.35 ± 1.24 mg/100 g TGH (TGP-4), 67.88 ± 1.33 mg/100 g TGH (TGP-5), and 5.93 ± 0.61 mg/100 g TGH (TGP-6), respectively (Table 1). 

### 2.2. Amino Acid Sequence Analysis and Mass Spectrometry of APs (TGP1–TGP6)

The amino acid sequences and molecular mass of six APs (TGP1–TGP6) were determined using a protein sequencer and electrospray ionization (ESI)-mass spectrometer (MS), and the data are shown in Table 1. The amino acid sequences of six APs (TGP1–TGP6) were identified as Glu-Pro-Leu-Ser-Asp (EPLSD, TGP-1), Trp-Ile-Asp-Pro-Asp-Gly (WLDPDG, TGP-2), Met-Asp-Leu-Phe-Thr-Glu (MDLFTE, TGP-3), Trp-Pro-Pro-Asp (WPPD, TGP-4), Glu-Pro-Val-Val (EPVV, TGP-5), and Cys-Tyr-Ile-Glu (CYIE, TGP-6) with MWs of 559.55, 701.69, 754.81, 513.50, 442.48, and 526.57 Da, respectively, which were in well accordance with their theoretical masses (Table 1).

### 2.3. Antioxidant Activity

**Figure 5:**
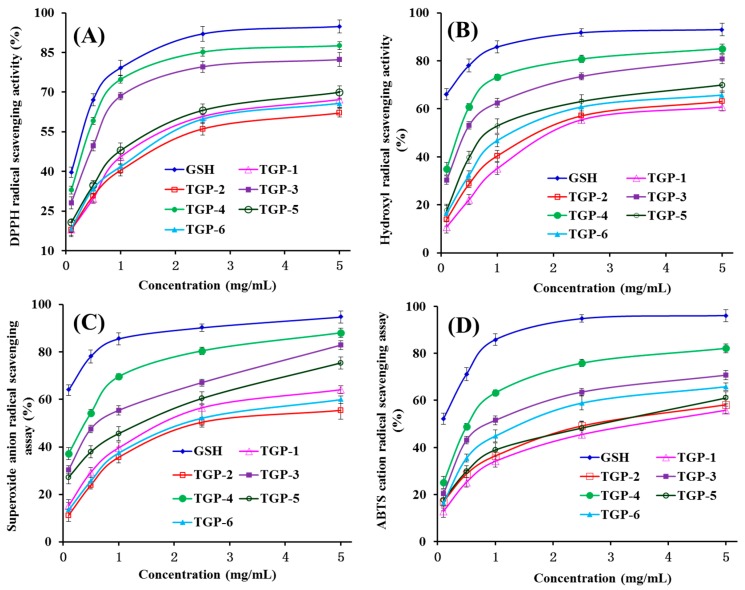
DPPH (**A**), hydroxyl (**B**), superoxide anion (**C**), and ABTS cation (**D**) radical scavenging activities of six APs (TGP1–TGP6) from protein hydrolysate of blood cockle (*T. granosa*). All data are expressed as mean ± SD (n = 3).

**Figure 6:**
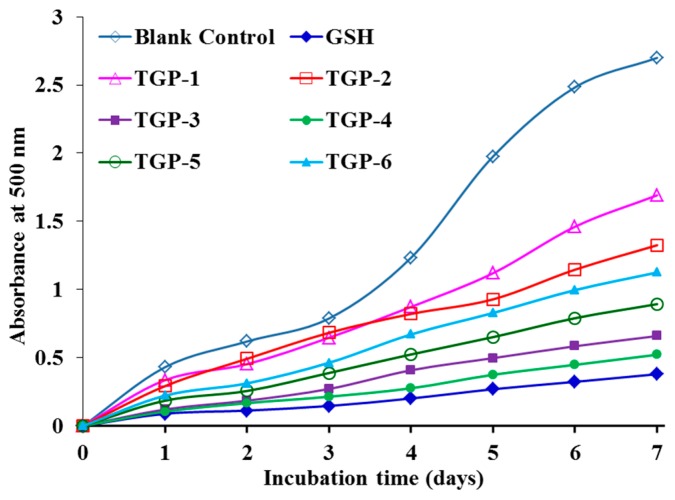
Lipid peroxidation inhibition assays of six APs (TGP1–TGP6) from protein hydrolysate of blood cockle (*T. granosa*). All data are expressed as mean ± SD (n = 3).

To better evaluate the activity of six APs (TGP1–TGP6) from blood cockle (*T. granosa*), four kinds of radical scavenging assays and lipid peroxidation inhibition assay were employed, and the data were presented in Table 2 and Figure 5 and Figure 6.

#### 2.3.1. Radical Scavenging Activity

##### DPPH Radical Scavenging Activity

As shown in Figure 5A, six APs (TGP1–TGP6) showed strong DPPH radical scavenging activities with a positive correlation between the activity and the concentration, but TGP1–TGP6 still showed weaker activity than the positive control of GSH did at the same concentration. EC_50_ values of TGP3 and TGP4 were 0.53 ± 0.02 mg/mL and 0.36 ± 0.02 mg/mL, respectively, which were significantly lower than those of TGP-1 (1.23 ± 0.09 mg/mL), TGP-2 (1.82 ± 0.16 mg/mL), TGP-5 (1.13 ± 0.14 mg/mL), and TGP-6 (1.30 ± 0.11 mg/mL), respectively. In addition, the EC_50_ values of TGP3 and TGP4 were lower than those of APs from protein hydrolysates of miiuy croaker muscle (FWKVV: 0.85 mg/mL; FMPLH: 0.48 mg/mL) [2] and swim bladder (GIEWA: 0.78 mg/mL) [26], blue mussel (YPPAK: 2.62 mg/mL) [27], and skipjack tuna bone (GADIVA: 0.57 mg/mL) [16]. However, the EC_50_ values of TGP3 and TGP4 were higher than those of APs from protein hydrolysates of skipjack tuna bone (GAEGFIF: 0.30 mg/mL) [16], skate muscle (NWDMEKIWD: 0.289 mg/mL) [28], bluefin leatherjacket skin (FIGP: 0.118 mg/mL) [29], and grass carp skin (HFGBPFH: 0.20 mg/mL) [30], respectively. DPPH is popularly employed to evaluate the antioxidant ability of APs due to its cell-permeable and stable properties. Therefore, six APs (TGP1–TGP6), especially TGP3 and TGP4 had the strong ability to serve as hydrogen donors or free radical scavengers for preventing the chain reaction of DPPH radical.

##### Hydroxyl Radical Scavenging Activity

Figure 5B indicated that six APs (TGP1–TGP6) showed concentration-related efficiency in the scavenging activity of hydroxyl radical at concentrations ranging between 0.1 and 5.0 mg/mL. EC_50_ values of TGP3 (0.47 ± 0.03 mg/mL) and TGP4 (0.38 ± 0.04 mg/mL) were significantly (*p* < 0.05) lower than those of TGP1 (2.18 ± 0.16 mg/mL), TGP2 (1.54 ± 0.11 mg/mL), TGP5 (1.09 ± 0.08 mg/mL), and TGP6 (1.29 ± 0.13 mg/mL), respectively. However, the scavenging activity of TGP1–TGP6 was still lower than that of the positive control (GSH). EC_50_ values of TGP3 and TGP4 were lower than those of APs from protein hydrolysates of miiuy croaker muscle (FWKVV: 0.97 mg/mL; FMPLH: 0.80 mg/mL) [2] and swim bladder (FPYLRH: 0.68 mg/mL; GIEWA: 0.71 mg/mL) [26], grass carp skin (PYSFK: 2.283 mg/mL; VGGRP: 2.055 mg/mL) [30], weatherfish loach (PSYV: 2.64 mg/mL) [31], hairtail muscle (KA: 1.740 mg/mL; AKG: 2.378 mg/mL; IYG: 2.498 mg/mL) [32], giant squid (NADFGLNGLEGLA: 0.612 mg/mL) [33], and conger eel (LGLNGDDVN: 0.687 mg/mL) [34]. However, EC_50_ values of TGP3 and TGP4 were higher than those of APs from skipjack tuna bone (GADIVA: 0.25 mg/mL; GAEGFIF: 0.32 mg/mL) [16], monkfish muscle (EWPAQ: 0.269 mg/mL; FLHRP: 0.114 mg/mL; LMGQW: 0.040 mg/mL) [35], croceine croaker scale (GFRGTIGLVG: 0.293 mg/mL, GPAGPAG: 0.240 mg/mL, and GFPSG: 0.107 mg/mL) [6], and spotless smoothhound (*Mustelus griseus*) muscle (GIISH: 0.0769 mg/mL; ELLI: 0.1173 mg/mL; KFPE: 0.1510 mg/mL) [36]. Hydroxyl radicals can initiate the oxidative stress process through promptly attacking and indiscriminately oxidizing biomacromolecules in an organism. The data indicated that TGP3 and TGP4 could serve as scavengers for weakening the biological system damage of hydroxyl radical. 

##### Superoxide Anion Radical Scavenging Activity

Figure 5C indicated six APs (TGP1–TGP6) strongly scavenge superoxide anion radical with a concentration-related efficiency manner at a concentration ranging 0.1–5.0 mg/mL, but their scavenging activities were still lower than that of GSH. EC_50_ values of TGP1–TGP6 were 2.04 ± 0.23, 2.49 ± 0.17, 0.75 ± 0.04, 0.46 ± 0.05, 1.69 ± 0.14, and 2.31 ± 0.15 mg/mL, respectively. EC_50_ values of TGP3 and TGP4 were lower than those of APs from protein hydrolysates of miiuy croaker muscle (NFWWP: 0.84 mg/mL; YFLWP: 3.08 mg/mL) [2] and swim bladder (GFEPY: 0.87 mg/mL; FTGMD: 3.04 mg/mL; FSGLR: 3.35 mg/mL) [26], skipjack tuna bone (GPDGR: 1.44 mg/mL; AGPM: 1.68 mg/mL) [16], and hairtail (*Trichiurus japonicas*) muscle (KA: 2.082 mg/mL; AKG: 2.538 mg/mL; IYG: 1.355 mg/mL) [29]. However, EC_50_ values of TGP3 and TGP4 were higher than those of APs from protein hydrolysates of miiuy croakers muscle (FWKVV: 0.29 mg/mL; FMPLH: 0.15 mg/mL) [2] and swim bladder (FPYLRH: 0.34 mg/mL; GIEWA: 0.30 mg/mL) [26], monkfish muscle (FLHRP: 0.101 mg/mL; LMGQW: 0.042 mg/mL) [35], and croceine croaker scale (GPAGPAG: 0.099 mg/mL; GFPSG: 0.151 mg/mL) [6]. Superoxide anion radical can produce hydroxyl radical to cause oxidative stress because it and its metabolites can initiate lipid peroxidation, react with carbonyl compounds for producing toxic peroxy radicals, and inactivate enzyme activity. Under normal physiological conditions, superoxide dismutase (SOD) catalyzes the dismutation of superoxide anions into hydrogen peroxide and oxygen [37,38,39]. Therefore, TGP3 and TGP4 can assist SOD to reduce superoxide anion radical damage in organisms.

##### ABTS Cation Radical Scavenging Activity

As shown in Figure 5D, the ABTS cation radical scavenging rates of six APs (TGP1–TGP6) increased with evaluated concentration ranging 0.1–5.0 mg/mL, but their activities were still lower than that of GSH at the same concentration. EC_50_ values of TGP3 and TGP4 were 0.96 ± 0.08 and 0.54 ± 0.03 mg/mL, respectively, which were significantly (*p* < 0.05) lower than those of TGP-1 (3.28 ± 0.17 mg/mL), TGP-2 (2.56 ± 0.23 mg/mL), TGP-5 (2.54 ± 0.17 mg/mL), and TGP-6 (1.86 ± 0.15 mg/mL), respectively. Furthermore, EC_50_ values of TGP3 and TGP4 were lower than those of APs from protein hydrolysates of skipjack tuna bone (GPDGR: 1.07 mg/mL; AGPM: 1.48 mg/mL) [16] and muscle (NFWWP: 0.84 mg/mL; YFLWP: 3.08 mg/mL) [2], skate cartilage (IVAGPQ: 1.29 mg/mL; FIMGPY: 1.04 mg/mL) [8], spotless smoothhound muscle (GAA 1.7541 mg/mL; GFVG 1.3055 mg/mL), and salmon (FLNEFLHV: 1.548 mg/mL) [40]. ABTS is popularly used to measure the capacities of APs, and the finding indicated that six APs (TGP1–TGP6), especially TGP3 and TGP4, could effectually inhibit the chain reaction of ABTS cation radicals by converting them to the colorless form.

#### 2.3.2. Lipid Peroxidation Inhibition Ability

As presented in Figure 6, the absorbance values of TGP3 and TGP4 solutions at 500 nm were significantly (*p* < 0.5) lower than those of other four APs (TGP1, TGP2, TGP5, and TGP6) and the blank control without antioxidant. However, the 500 nm absorbance values of TGP3 and TGP4 solutions were little greater than that of the positive control (GSH). The oxidative process is complicated in food and biological systems and embroiled in multifarious reactions for propagation of lipid radicals hydroperoxides [3,5]. Those data of TGP3 and TGP4 in the linoleic acid model system indicated that they have a strong ability to inhibit lipid peroxidation. In addition, these abilities of TGP3 and TGP4 were similar to that of GSH in the seven days incubation.

Molecular size plays a key role in the antioxidant capacities of APs [5,24]. Six APs (TGP1–TGP6) from protein hydrolysate of blood cockle (*T. granosa*) are tetrapeptide to hexapeptide with MWs ranging 442.48 Da–754.81 Da (Table 1). These data indicated that six APs could easily interact with free radicals to inhibit the lipid peroxidation. Furthermore, amino acid composition and sequence are believed to play major contributions to the activities of APs [5,29,36]. Hydrophobic amino acid residues, including aliphatic (Val, Leu, and Ile), aromatic (Phe, Trp, and Tyr), and sulfur-containing (Met and Cys) can play their functions on radical scavenging because of their high reactivity to hydrophobic PUFAs in lipid-rich foods [26,41]. Aromatic residues can donate protons to electron deficient radicals to keep ROS stable during the radical scavenging process [5]. Sulfur-containing amino acid residues (Met and Cys) might work as a reactive site, where the peptide could scavenge oxidants through the formation of a sulfoxide structure after oxidation to stop free-radical chain reactions [2,42,43,44]. Therefore, hydrophobic/aromatic amino acid residues in TGP3 (Met, Leu, and Phe) and TGP4 (Trp and Pro) should contribute to their activity through helping them to contact target radicals. Giménez et al. [45] and Zhu et al. [46] found that polar amino acid residues (Glu, Asp, and Lys) played a critical role in antioxidant activity including metal ion chelating and hydroxyl radical scavenging activities. Gly residue can make the peptide skeleton more flexible and its single hydrogen atom can serve as proton-donating to neutralize free radicals [47,48,49]. Therefore, polar amino acids including Asp and Glu residues in TGP3, and Asp residues in TGP4 could play a critical role in the lipid peroxidation inhibition activities.

### 2.4. Effects of Thermal, pH, and Simulated Gastrointestinal (GI) Digestion Treatments on TGP3 and TGP4 Stability

**Figure 7:**
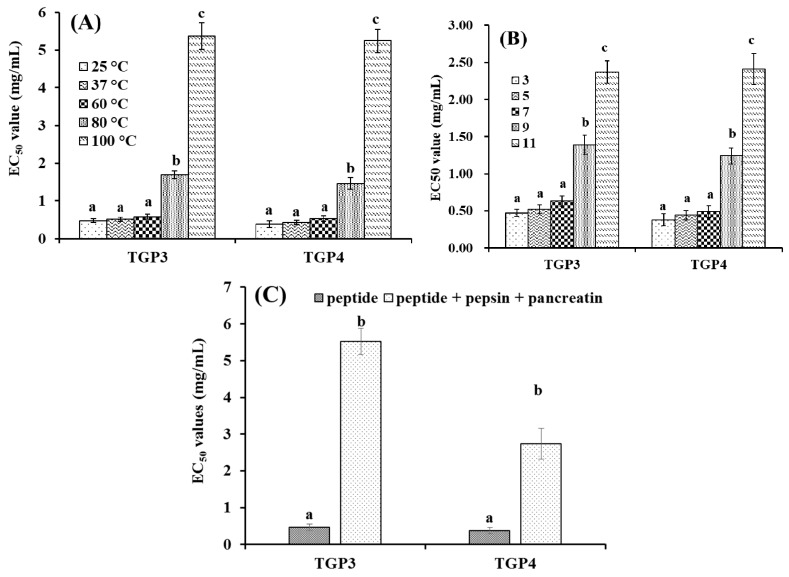
Hydroxyl radical scavenging activity of TGP3 and TGP4 subjected to heat treatments (**A**), pH treatments (**B**), and simulated GI digestion treatments (**C**). All data are expressed as mean ± SD (n = 3). a–c values with same letters indicate no significant difference of same sample (*p* > 0.05).

As shown in Figure 7A, heat treatments could influence the hydroxyl radical scavenging activity (expressed as EC_50_) of TGP3 and TGP4. When TGP3 and TGP4 were treated at 25, 37, and 60 °C for 0.5 h, their EC_50_ values on hydroxyl radical did not show a significant (*p* > 0.05) difference, but were significantly (*p* < 0.05) lower than those of sample treated at 80 and 100 °C for 0.5 h.

The EC_50_ values of TGP3 and TGP4 on hydroxyl radical treated at pH 3 to 11 were presented in Figure 7B. There were no significant (*p* > 0.05) differences when the pH changed from 3 to 7. However, EC_50_ values of TGP3 and TGP4 on hydroxyl radical in acidic and neutral conditions were significantly (*p* < 0.05) lower than those of alkaline conditions (pH 9 to 11).

In response to simulated GI digestion, the hydroxyl radical scavenging activities of TGP3 and TGP4 are shown in Figure 7C. The EC_50_ values for TGP3 and TGP4 before simulated GI digestion (TGP3: 0.47 ± 0.03 mg/mL; TGP4: 0.38 ± 0.04 mg/mL) were significantly (*p* < 0.05) lower than those obtained after simulated GI digestion (TGP3: 5.52 ± 0.36 mg/mL; TGP4: 2.74 ± 0.42 mg/mL).

Heat treatment is a common method of food processing and APs are helpful to lengthen the food shelf-life if they can keep their activity after heating. Peptides with large-scale pH stability can be incorporated into diverse liquid products and keep their bioactivity. Therefore, thermal and pH stability of peptides are important indexes for their application in functional products, and characterization of those properties can help to design their potential processing parameters [15,50]. ATSHH from protein hydrolysate of sandfish (*Arctoscopus japonicus*) partially lost its DPPH radical scavenging activity when it was incubated at 50–90 °C. In addition, ATSHH bored moderate losses of activity under basic (pH 10–12) and acidic (pH 2) conditions [50]. However, there are no significant (*p* > 0.05) differences when WAFAPA and MYPGLA from blue-spotted stingray are incorporated during heat (25–100 °C) and pH (3–11) treatments [15]. In the experiment, EC_50_ values of TGP3 and TGP4 on hydroxyl radical significantly (*p* < 0.005) increased at temperatures above 80 °C and pH values higher than 9, which indicated that TGP3 and TGP4 were not suitable for high temperature treatment and basic (pH > 9.0 for 2.5 h) food products. The capacity of TGP3 and TGP4 to resist GI digestion is one of the key requirements for their applications in vivo, which may tell whether they will be used as food ingredients or nutraceuticals in isolated forms. Then, simulated GI digestion is usually used to evaluate the fate of peptides before exploring their bioactivity and bioavailability in vivo [50]. In this assay, EC_50_ values of TGP3 and TGP4 with simulated GI digestion on hydroxyl radical were significantly (*p* < 0.05) increased (Figure 7C), which reflected that TGP3 and TGP4 are partially susceptible to be degradated by GI digestive enzymes. In addition, Figure 7C shows that the effect of GI digestion on TGP4 was significantly weaker than that on TGP3. The results were in accordance with the report by Segura-Campos et al. that bioactive peptides containing Pro residues generally stand up to degradation by GI digestive enzymes [51]. Taken together, TGP3 and TGP4 are unstable under high thermal food processing and cannot retain bioactivity under basic pH conditions.

## 3. Experimental Section

### 3.1. Materials

Blood cockle (*T. granosa*) was purchased from Fengmao market (Zhoushan, China) and authenticated by Professor Sheng-long Zhao (Zhejiang Ocean University, Zhoushan, China). Sephadex G-25, Sephadex G-15, GSH, bovine serum albumin (BSA), and DEAE-52 cellulose were purchased from Shanghai Source Poly Biological Technology Co., Ltd. (Shanghai, China). DPPH, phosphate buffered saline (pH 7.2), 2,4,6-trinitrobenzenesulfonic acid solution (TNBS), and ABTS were bought from Sigma-Aldrich (Shanghai) Trading Co., Ltd. (China). Acetonitrile (LC grade) and trifluoroacetic acid (TFA) were purchased from Thermo Fisher Scientific Co., Ltd. (Shanghai, China). Six APs (TGP1 to TGP6) with purity of 98% were synthesized in Shanghai Apeptide Co. Ltd. (Shanghai, China).

### 3.2. Preparation of Protein Hydrolysate (TGH) of Blood Cockle (T. granosa)

The defatting process of blood cockle was carried out according to the previous methods [2,17]. Blood cockle internal organs were removed, and the resulting meat was rinsed and homogenized using a JJ-2 Kinematica (Jiangsu Jiangling Co., Ltd., Yancheng, China). The homogenate and isopropanol were mixed in a ratio of 1:4 (*w*/*v*) and stirred uninterrupted at 35 °C for 2.5 h, and the defatting process was performed three times. After that, the degreasing mixture was centrifuged at 9000 *g* for 20 min at 4 °C. The supernatant was removed, and the sediment was freeze-dried and stored at −20 °C.

The hydrolysis process was carried out using a two-enzyme system (Alcalase treatment for 1.5 h following Neutrase treatment for 1.5 h). The defatted precipitate (100 g) was dissolved (5%, *w*/*v*) in 0.05 M Tris–HCl buffer solution (pH 8.5) and hydrolyzed using Alcalase at 50.0 °C with enzyme dose 1.5% (*w*/*w*) for 1.5 h. After that, the pH of dispersions was changed with HCl solution (1.0 M) and hydrolyzed using Neutrase at pH 7.0, 55.0 °C with enzyme dose 1.5% (*w*/*w*) for 1.5 h. Afterwards, the hydrolysate was kept in a 95 °C water bath for 10 min to inactivate proteases and centrifuged at 12,000 *g* for 15 min. The resulted supernatant, referred to as TGH, was freeze-dried and stored at −20 °C. 

The concentrations of TGH and hydrolysate fractions were expressed as mg protein/mL and determined by the dye binding method of Bradford (1976) with BSA as the standard protein [52].

### 3.3. Isolation of APs from TGH

#### 3.3.1. Fractionation of TGH

TGH was fractionated by ultrafiltration using Labscale TFF System of Millipor Ltd. (Billerica, MA, USA) with 3, 5, and 10 kDa MWCO membranes, and four fractions, termed as TGH-І (<3 kDa), TGH-II (3–5 kDa), TGH-III (5–10 kDa), and TGH-IV (>10 kDa), were collected and freeze-dried.

#### 3.3.2. Chromatography Isolation of APs from TGH-I

10 mL of TGH-I solution (40.0 mg protein/mL) was added into a DEAE-52 cellulose column (2.0 × 100 cm) pretreated with DW, and stepwise eluted with 150 mL DW, 0.1 M NaCl, 0.5 M NaCl, and 1.0 M NaCl solution at a flow rate of 1.0 mL/min, respectively. Each eluate (5 mL) was monitored at 214 nm. Finally, six fractions (TGH-I-1 to TGH-I-6) were pooled on chromatographic peak and freeze-dried.

5 mL of TGH-I-5 solution (30.0 mg protein/mL) was loaded onto a Sephadex G-25 column (2.5 × 200 cm) eluted with DW at a flow rate of 0.8 mL/min. Each eluate (3 mL) was collected and monitored at 214 nm, and TGH-I-5B solution (5 mL, 25.0 mg protein/mL) was further separated by a Sephadex G-15 column (2.0 × 180 cm) at a flow rate of 0.6 mL/min. Each eluate (3 mL) was collected and monitored at 214 nm, and four subfractions (TGH-I-5B1, TGH-I-5B2, TGH-I-5B3, and TGH-I-5B4) were collected and lyophilized.

Finally, TGH-I-5B3 was purified on an Agilent 1260 HPLC system (Agilent Ltd., Santa Rosa, CA, USA) with a Zorbax C-18 column (4.6 × 250 mm). The sample was eluated at a flow rate of 0.8 mL/min with a linear gradient of acetonitrile from 0% to 50% in 0–25 min in 0.1% TFA. Six APs (TGP1 to TGP6) were isolated on 214 nm absorbance and lyophilized.

### 3.4. Degree of Hydrolysis (DH)

DH analysis was performed according to the previously described method [6]. The hydrolysate (50μL) was mixed with 0.5 mL of 0.2 M phosphate buffered saline (PBS), pH 8.2 and 0.5 mL of 0.05% 2,4,6-trinitrobenzene sulfonic acid (TNBS) reagent. TNBS was freshly prepared before use by diluting with DW water. The mixture was incubated at 50 °C for 1 h in a water bath. The reaction was stopped by adding 1 mL of 0.1 M HCl and incubated at room temperature for 30 min. The absorbance was monitored at 420 nm. l-Leucine was used as a standard. To determine the total amino acid content, TGH was completely hydrolyzed with 6 M HCl with a sample to acid ratio of 1:100 at 120 °C for 24 h. DH (%) was calculated using the following equation:

DH = [(A_t_ − A_0_)/(A_max_ − A_0_)] ×100 (1)

where A_t_ is the amount of a-amino acids released at time t, A_0_ is the amount of amino acids in the supernatant at 0 h, and Amax is the total amount of a-amino acids obtained after acid hydrolysis at 120 °C for 24 h.

### 3.5. Antioxidant Activity

#### 3.5.1. Radical Scavenging Activity

The DPPH radical, hydroxyl radical, superoxide anion radical, and ABTS cation radical scavenging activities were measured according to the previous methods [6,53,54]. The results were expressed as a half elimination ratio (EC_50_) defined as the concentration by which a sample caused a 50% decrease of the initial concentration of DPPH radical, hydroxyl radical, superoxide anion radical, and ABTS cation radical, respectively, and the calculation method of EC_50_ was according to linear relationship of radical scavenging rates and concentrations of samples [6,26].

##### DPPH Radical Scavenging Activity

Two millilitres of samples consisting of DW and different concentrations of the analytes were placed in cuvettes, and 500 μL of an ethanolic solution of DPPH (0.02%) and 1.0 mL of ethanol were added. A control sample containing the DPPH solution without the sample was also prepared. In the blank, the DPPH solution was substituted with ethanol. The antioxidant activity of the sample was evaluated using the inhibition percentage of the DPPH radical with the following equation:

DPPH radical scavenging activity (%) = (A_0_ + A_b_ − A)/A_0_ × 100% (2)

where A is the absorbance rate of the sample, A_0_ is the control group absorbance, and A_b_ is the blank absorbance.

##### Hydroxyl Radical Scavenging Activity

First, 1.0 mL of a 1.87 mM 1,10-phenanthroline solution and 2.0 mL of the sample were added to a screw-capped tube and mixed. Then, 1.0 mL of a FeSO_4_·7H_2_O solution (1.87 mM) was added to the mixture. The reaction was initiated by adding 1.0 mL of H_2_O_2_ (0.03%, *v*/*v*). After being incubated at 37 °C for 60 min in a water bath, the absorbance of the reaction mixture was measured at 536 nm against a reagent blank. The reaction mixture without any antioxidant was used as the negative control, and a mixture without H_2_O_2_ was used as the blank. The hydroxyl radical scavenging activity was calculated using the following formula:

Hydroxyl radical scavenging activity (%) = [(A_s_ − A_n_)/(A_b_ − A_n_)] × 100% (3)

where A_s_, A_n_, and A_b_ are the absorbance values determined at 536 nm of the sample, the negative control, and the blank after the reaction, respectively.

##### Superoxide Anion Radical Scavenging Activity

The superoxide anions were generated in 1 mL of nitrotetrazolium blue chloride (2.52 mM), 1 mL of nicotinamide adenine dinucleotide hydride (NADH) (624 mM) and 1 mL of different sample concentrations. The reaction was initiated by adding 1 ml of phenazine methosulphate solution (120 μM) to the reaction mixture. The absorbance was measured at 560 nm against the corresponding blank after 5 min incubation at 25 °C. The superoxide anion radical scavenging capacity was calculated using the following equation:

Superoxide anion radical scavenging activity (%) = [(A_control_ − A_sample_)/A_control_] × 100% (4)

where A_control_ is the absorbance without sample and A_sample_ is the absorbance with sample.

##### ABTS Cation Radical Scavenging Activity

The ABTS radical cation was generated by mixing ABTS stock solution (7 mM) with potassium persulphate (2.45 mM). The mixture was left in the dark at room temperature for 16 h. The ABTS radical solution was diluted in 5 mM PBS pH 7.4, to an absorbance of 0.70 ± 0.02 at 734 nm. One milliliter of diluted ABTS radical solution was mixed with one milliliter of different concentrations of samples. 10 min later, the absorbance was measured at 734 nm against the corresponding blank. The ABTS scavenging activity of samples was calculated using the following equation:

ABTS scavenging activity (%) = [(A_control_ − A_sample_)/A_control_] × 100% (5)

where A_control_ is the absorbance without sample and A_sample_ is the absorbance with sample.

#### 3.5.2. Lipid Peroxidation Inhibition Assay

The lipid peroxidation inhibition and radical scavenging assays of TGP1 to TGP6 were measured according to the previous method [6,55]. In brief, a sample (5.0 mg) was dissolved in 10 mL of 50 mM phosphate buffer (pH 7.0), and added to a solution of 0.13 mL of linoleic acid and 10 mL of 99.5% ethanol. Then, the total volume was adjusted to 25 mL with DW. The mixture was incubated in a conical flask with a screw cap at 40 °C in a dark room and the degree of oxidation was evaluated by measuring the ferric thiocyanate values. The reaction solution (100 μL) incubated in the linoleic acid model system was mixed with 4.7 mL of 75% ethanol, 0.1 mL of 30% ammonium thiocyanate, and 0.1 mL of 20 mM ferrous chloride solution in 3.5% HCl. After 3 min, the thiocyanate value was measured by reading the absorbance at 500 nm following color development with FeCl_2_ and thiocyanate at different intervals during the incubation period at 40 °C.

### 3.6. Amino Acid Sequence and Molecular Mass Analysis

Amino acid sequences and molecular masses of TGP1 to TGP6 were measured on the previous method [26,56]. TGP1 to TGP6 were subjected to N-terminal amino acid sequencing on an Applied Biosystems 494 protein sequencer (Perkin Elmer/Applied Biosystems Inc., Foster City, CA, USA). Edman degradation was performed according to the standard program supplied by Applied Biosystems. Accurate molecular masses of TGP1 to TGP6 were determined using a Q-TOF mass spectrometer (Micromass, Waters, Milford, MA, USA) coupled with an ESI source. 

### 3.7. Stability Properties of TGP3 and TGP4 against Heat, pH, and Simulated GI Digestion Treatments

Stability of TGP3 and TGP4 were measured according to the previous method with minor modifications [49]. A temperature-controlled water bath at 25, 37, 60, 80, or 100 °C for 0.5 h was used to measure thermostability of TGP3 and TGP4. Effects of pH treatments (pH 3, 5, 7, 9, or 11) of sample solutions incubated at 25 °C for 2.5 h were assessed to analyze the pH stability of TGP3 and TGP4. A two-stage digestion model (pepsin for 1.0 h + pancreatin for 2.0 h) was applied to simulate GI digestion of TGP3 and TGP4. Hydroxyl radical scavenging activities (EC_50_ value) of the treated TGP3 and TGP4 were measured according to the previous methods [6,26]. 

### 3.8. Statistical Analysis

The data are expressed as the mean ± SD (n = 3). A one-way analysis of variance (ANOVA) test for differences between means of each group was applied to analyze data using SPSS 19.0 (Statistical Program for Social Sciences, SPSS Corporation, Chicago, IL, USA). A *P*-value of less than 0.05 was considered statistically significant. 

## 4. Conclusions

In the experiment, blood cockle (*T. granosa*) was hydrolyzed under a two-enzyme system (Alcalase treatment for 1.5 h following Neutrase treatment for 1.5 h) and six APs (TGP1–TGP6) were isolated from the resulting hydrolysate (TGH) and identified as EPLSD (TGP1), WLDPDG (TGP2), MDLFTE (TGP3), WPPD (TGP4), EPVV (TGP5), and CYIE (TGP6), respectively. Six APs (TGP1–TGP6), especially TGP3 and TGP4, exhibited high radical scavenging and lipid peroxidation inhibition capabilities. However, TGP3 and TGP4 are unstable and cannot retain antioxidant activity at high temperatures (>80 °C for 0.5 h), basic pH conditions (pH > 9 for 2.5 h), or during simulated GI digestion. Therefore, TGP3 and TGP4 may be more suitable to serve as nutraceutical candidates in isolated forms than as food ingredient candidates. In addition, in vivo experiments to elucidate the antioxidant mechanisms of the six APs (TGP1–TGP6) need to be performed in future.

## Figures and Tables

**Table 1:** Retention time (min), yields (mg/100 g TGH), amino acid sequences, and molecular masses (Da) of six APs (TGP1-TGP6) from protein hydrolysate of blood cockle (*T. granosa*).

No.	Retention Time (min)	Yield (mg/100 g TGH)	Amino Acid Sequence	Theoretical Mass/Observed Mass (Da)
TGP-1	8.586	40.52 ± 3.76	Glu-Pro-Leu-Ser-Asp (EPLSD)	559.57/559.55
TGP-2	9.831	9.43 ± 0.64	Trp-Ile-Asp-Pro-Asp-Gly (WLDPDG)	701.72/701.69
TGP-3	10.679	23.46 ± 1.51	Met-Asp-Leu-Phe-Thr-Glu (MDLFTE)	754.85/754.81
TGP-4	12.394	12.35 ± 1.24	Trp-Pro-Pro-Asp (WPPD)	513.54/513.50
TGP-5	12.692	67.88 ± 1.33	Glu-Pro-Val-Val (EPVV)	442.51/442.48
TGP-6	13.762	5.93 ± 0.61	Cys-Tyr-Ile-Glu (CYIE)	526.60/526.57

The yields of six APs (TGP1–TGP6) are expressed as mean ± SD (n = 3).

**Table 2:** Radical scavenging activity of six isolated APs (TGP1–TGP6) from protein hydrolysate of blood cockle (*T. granosa*).

No.	Half Elimination Ratio (EC_50_, mg/mL)			
	DPPH Radical	Hydroxyl Radical	Superoxide Anion Radical	ABTS Cation Radical
**TGP-1**	1.23 ± 0.09 ^a,b^	2.18 ± 0.16 ^a^	2.04 ± 0.23 ^a^	3.28 ± 0.17 ^a^
**TGP-2**	1.82 ± 0.16 ^c^	1.54 ± 0.11 ^b^	2.49 ± 0.17 ^b^	2.56 ± 0.23 ^b^
**TGP-3**	0.53 ± 0.02 ^d^	0.47 ± 0.03 ^c^	0.75 ± 0.04 ^c^	0.96 ± 0.08 ^c^
**TGP-4**	0.36 ± 0.02 *^e^*	0.38 ± 0.04 ^c^	0.46 ± 0.05 ^d^	0.54 ± 0.03 ^d^
**TGP-5**	1.13 ± 0.14 ^a^	1.09 ± 0.08 ^d^	1.69 ± 0.14 ^e^	2.54 ± 0.17 ^b^
**TGP-6**	1.30 ± 0.11 ^b^	1.29 ± 0.13 ^b^	2.31 ± 0.15 ^b^	1.86 ± 0.15 ^e^

All data are expressed as mean ± SD (n = 3). ^a–e^ Values with same letters indicated no significant difference of different sample at same radicals (*p* > 0.05).
